# Orthodontic treatment in the presence of aggressive periodontitis

**DOI:** 10.1590/2177-6709.26.6.e21bbo6

**Published:** 2021-12-17

**Authors:** Alexandre Trindade Simões da MOTTA

**Affiliations:** 1 Universidade Federal Fluminense, Department of Orthodontics (Niterói/RJ, Brazil).; 2 Private practice (Rio de Janeiro/RJ, Brazil).

**Keywords:** Aggressive periodontitis, Corrective Orthodontics, Crossbite, Dental implantation

## Abstract

**Introduction:**

Aggressive periodontitis causes periodontal destruction, with loss of supporting alveolar bone. The common symptom is rapid attachment loss in the first molar and incisor area, in young adults.

**Objective:**

The aim of this study was to discuss the challenges, implications and the impact of orthodontic treatment in patients affected by severe periodontal problems, specifically aggressive periodontitis.

**Discussion:**

In addition to other bacteria, the main pathogen involved in aggressive periodontitis is the *Aggregatibacter actinomycetemcomitans*. However, the susceptibility to the disease differs among individuals, being immune deficiencies the main reason for this variability. Many orthodontists are not comfortable about performing treatments on individuals with aggressive periodontitis.

**Conclusion:**

Orthodontic treatment is feasible in young patients with severe and localized aggressive periodontitis, as long as the limitations imposed by the disease are respected. An interdisciplinary approach is required, with frequent periodontal follow-up before, during and after orthodontic treatment, allowing the correction of dental positions without aggravating bone loss.

## RESUMO

**Introdução:**

A periodontite agressiva causa destruição periodontal, com perda do osso alveolar de suporte. O sintoma mais comum é a rápida perda de inserção na área de primeiros molares e incisivos, em adultos jovens.

**Objetivo:**

O objetivo do presente trabalho foi discutir os desafios, as implicações e os impactos do tratamento ortodôntico em pacientes acometidos por problemas periodontais graves, especificamente a periodontite agressiva.

**Discussão:**

Apesar da presença de outras bactérias, o principal patógeno envolvido na periodontite agressiva é o *Aggregatibacter actinomycetemcomitans*. Entretanto, a suscetibilidade da doença difere entre os indivíduos, sendo as deficiências imunológicas o principal motivo dessa variabilidade. Muitos ortodontistas não se sentem confortáveis em realizar tratamentos em indivíduos com periodontite agressiva.

**Conclusão:**

O tratamento ortodôntico é possível em pacientes jovens com periodontite agressiva severa e localizada, desde que se respeite as limitações impostas pela doença. Uma abordagem multidisciplinar é fundamental, com acompanhamento periodontal frequente antes, durante e depois do tratamento ortodôntico, permitindo a correção dos posicionamentos dentários sem agravar a perda óssea.

## INTRODUCTION

Aggressive periodontitis (AgP) is a type of periodontitis with early onset and rapid progression, causing periodontal destruction, with loss of supporting alveolar bone.^1^ This disease causes localized breakdown of the periodontal attachment in specific regions of the dental arch early in life. The common symptom is rapid attachment loss in the first molar and incisor area.^2^

It mostly affects young adults in the age range of 15-35 years, which is the common age group for patients’ seeking orthodontic treatment. Therefore, education regarding periodontitis, especially AgP, is essential among orthodontists and general dentists.^3^

The *Aggregatibacter actinomycetemcomitans* (previously known as *Actinobacillus actinomycetemcomitans*) is found in high frequency in microbial deposits on the affected teeth, and is considered, besides other bacteria, the major pathogen to be involved in AgP. Even though, the susceptibility of the disease differs among individuals, being immune defects the reason for this variability.^1^

The prevalence of AgP varies considerably between studies. A systematic review found a relatively high prevalence in Africa and South America, compared with Europe, Asia and North America. However, the authors highlighted the weakness of the definition of this form of periodontal disease, and suggested that studies with less heterogeneity are needed.^4^ These findings show that AgP is a significant health problem in certain populations.^5^

Different forms of periodontal disease have a distinct impact on the quality of life. Patients with a diagnosis of generalized forms of chronic or aggressive periodontitis showed poorer quality of life than those diagnosed with localized AgP, which was shown mainly by significant differences in the physical pain and psychological discomfort.^6^

Many orthodontists are not comfortable in performing treatments in subjects with AgP.^7^ Otherwise, orthodontic treatment is feasible in young patients with severe and localized AgP, as orthodontic tooth movements are possible independent of the attachment level, and without worsening it.^8^ When treating periodontal patients, orthodontists must consider an interdisciplinary approach, since interaction with the periodontist and a proper chronology of events are important factors for success.^9^

These cases should be planned individually, considering bone losses suffered by each patient. Respecting some limitations, it is possible to improve the level of bone insertion and facilitate oral hygiene through the orthodontic treatment of adult patients with little bone support.^10^ It may also help to prevent inflammation and the recurrence of periodontal disease.^2^

Thus, the aim of this study is to discuss the challenges of orthodontic treatment in patients with severe periodontal problems. Additionally, it aims at reporting the clinical case of a young woman presenting AgP, with several dental and orthodontic problems related to it.

## DIAGNOSIS

Female patient, aged 16 years and 3 months, was referred by a periodontist for orthodontic treatment. She reported, in addition to the gingival problem, chief complaints about *“teeth shifting forward”*, *“teeth opening spaces”* and *“inverted bite on the back”*. She presented good general health condition, but with a family history of periodontal disease.

**Figure 1:**
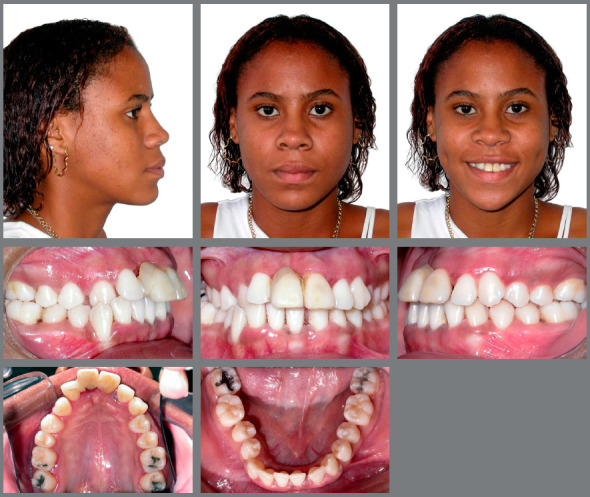
Pretreatment extraoral and intraoral photographs.

The frontal extraoral clinical examination revealed a slight deviation of the chin to the right, and an increased lower anterior facial height. The profile was convex, with a small nose, prominent chin, and protruded lips. The intraoral examination showed an Angle Class I with right anterior (#13) and posterior crossbite, a steep Curve of Spee, abnormal maxillary and mandibular arch forms, and deviated dental midlines (5mm) - upper to the left and lower to the right. An accentuated projection of the maxillary incisors (overjet = 8mm) was observed, probably resulting from periodontal collapse, mainly of the maxillary left central (#21) and maxillary right lateral (#12) incisors, which supported a provisional crown of the maxillary right central incisor (#11). Mandibular incisors were retroclined and had diastemas resulting from periodontal collapse, the mandibular right first molar (#46) was buccally displaced, and the maxillary right and left second molars (#17 and #27) were contracted and mesially rotated. Deficiency in anterior gingival papillae compromised esthetics and gingival retraction was marked in some teeth (Fig 1).

**Figure 2:**
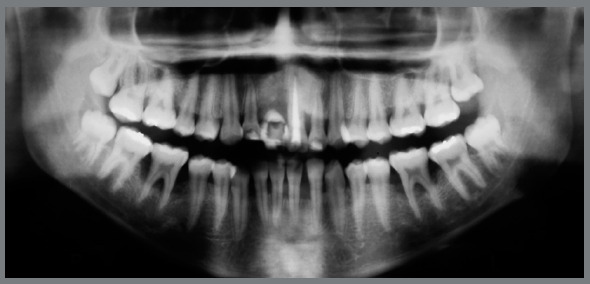
Pretreatment panoramic radiograph.

The radiographic examination revealed generalized bone loss, severe in some regions, such as the mandibular first molars (#36 and #46), with involvement of the furca, and in the maxillary and mandibular incisors, compatible with the diagnosis of aggressive periodontitis. The maxillary right central incisor (#11) had been lost due to lack of periodontal support, and the maxillary left central incisor (#21) had endodontic treatment and a history of endodontic lesion, with poor prognosis (Fig 2).

**Figure 3:**
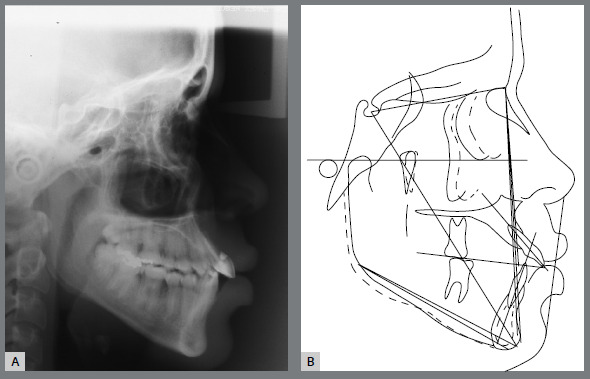
Pretreatment lateral cephalometric radiograph **(**A) and cephalometric tracing **(**B).

The cephalometric analysis revealed a skeletal Class I (ANB = 2^o^) with a negative Wits appraisal (-6.5 mm), showing a Class III tendency, and an increased vertical growth pattern (SN.GoGn = 38^o^; FMA = 30^o^). Dental measurements confirmed the marked projection of the maxillary incisors (1.NA = 37^o^; 1-NA = 13mm) and the retroclination of the mandibular incisors (IMPA = 79^o^) (Fig 3).

A functional analysis showed that lip sealing was not completely passive, due to upper incisors’ projection. Also, maxillary incisors showed a decreased exposure at rest, less than the ideal for a young woman. The enlarged shape of the mandibular arch indicated a large tongue with a low rest posture, but without the habit of projection. There was also a lack of adequate guides in the excursive mandibular movements, resulting from crossbite and other occlusal problems.

### TREATMENT OBJECTIVES

The treatment objectives of the present clinical case were initially based on preventing disease progression through frequent periodontal follow-up before, during and after orthodontic treatment, allowing the correction of dental positions without aggravating bone loss.

Regarding facial esthetics, the aim was to obtain facial symmetry, maintain the profile and vertical dimension, and increase the exposure of the maxillary incisors at smile. In dental aspects, the objective was to maintain Angle Class I, correct crossbite, arch form and level the curve of Spee; to obtain coincident midlines and a correct overjet, promoting space in the maxillary arch to reduce incisor projection and prepare the space for rehabilitation of the maxillary right central incisor (#11). Finally, to establish good intercuspation and adequate functional occlusal guides.

### TREATMENT OPTIONS

The considered treatment options were to perform posterior crossbite correction through conventional rapid maxillary expansion (RME), since the patient was 16 years old, or opt for a surgically-assisted maxillary expansion (SARME). At the time of treatment, the modality of miniscrew-assisted rapid palatal expansion (MARPE) was not yet established in the literature. It was then decided to try a RME with a conventional tooth-borne expander, without the aid of surgery, despite the borderline age.

### TREATMENT PROGRESS

**Figure 4:**
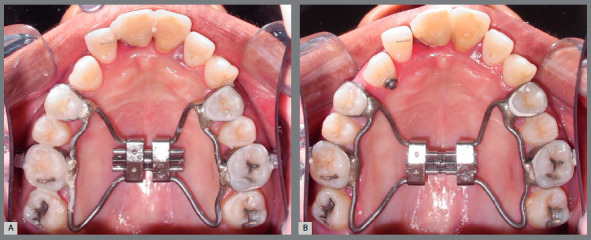
Maxillary arch occlusal views during maxillary expansion: A) placement of Hyrax expander; B) completion of screw activation.

Initially, third molar extraction surgery was performed. Then, a Hyrax expander was installed, with bands on the maxillary first molars (#16 and #26) and first premolars (#14 and #24), and distally extended to contour the palatal surface of the maxillary second molars (#17 and #27) to increase anchorage. The screw was activated twice a day for 14 days, totaling 7mm (Fig 4).

It was observed more dental than skeletal expansion, probably influenced by patient’s age and periodontal support, so the retention period for this procedure was reduced to only three months, then replacing bands with bonded accessories, facilitating hygiene. On the other hand, there was a significant opening of anterior spaces, which would favor the distalization of the maxillary right cuspid (#13) and correction of the upper midline.

**Figure 5:**
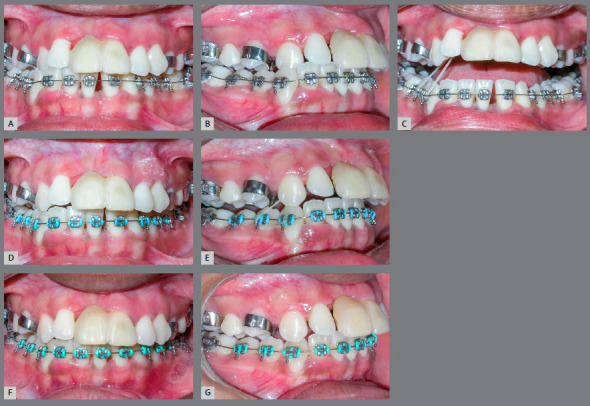
Maxillary right canine crossbite correction: Frontal **(**A) and right lateral**(**B) views of canine crossbite at the end of maxillary expansion. C) Frontal open mouth view of intermaxillary elastic in position. Frontal and right lateral views after 1 month **(D**, E) and 2 months **(F,** G) of cross elastics mechanics.

During the expansion retention, anterior crossbite correction was performed with a 1/8” intermaxillary elastic from the bracket hooks of mandibular right canine (#43) and first premolar (#44) to a bonded button on the palatal surface of the maxillary right cuspid (#13). A Roth prescription metallic fixed orthodontic appliance was mounted (0.022 x 0.028-in slot) with accessories initially bonded to all mandibular teeth, and the lower alignment and leveling was performed with 0.015-in Twist-flex, 0.014-in nickel-titanium (NiTi), 0.016-in and 0.018-in stainless steel archwires (Fig 5).

**Figure 6:**
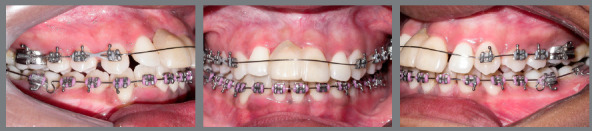
Alignment and leveling stage, and distalization of maxillary right canine with archwire bypassing maxillary incisors.

Subsequently, after correcting the crossbite of the maxillary right canine (#13) and removing the expander, the same type of appliance was mounted on the maxillary arch, but not including the incisors, to avoid its projection. Alignment and leveling was started with 0.015-in Twist-flex and 0.016-in NiTi archwires. Using spaces generated by the maxillary expansion, the distalization of the #13 was started with an elastic chain (Fig 6).

Maxillary incisor brackets were then bonded and included in the mechanics with the same 0.016-in NiTi archwire. Temporary tooth of the maxillary right central incisor (#11) was kept bonded to the mesial surface of maxillary left central incisor (#21) and in tie-together to the bracket of tooth #21, with metal ligature. A 0.019 x 0.025-in NiTi archwire was then placed, improving leveling, incisor inclination and filling the slot of the #11, providing greater stability to the temporary tooth. Then, a 0.018 x 0.025-in stainless steel archwire was inserted, continuing the distalization of the #13 to a Class I relationship. The space created mesial to the #13 would allow the upper midline correction to the right and a slight retraction of the maxillary incisors, thus reducing overjet.

In the mandibular arch, a 0.018 x 0.025-in stainless steel archwire was inserted and, using spaces present in the anterior region, correction of the midline to the left side was performed with an elastic chain. Then, a 0.019 x 0.026-in stainless steel archwire was inserted, constricted in the posterior region to help compensate the initial transverse problem. Performing interproximal reduction on mandibular incisors, small spaces were closed, thus reducing black spaces. The anchorage on this rectangular archwire was used for intermaxillary Class II elastics on the right side and anterior cross elastics, completing midline correction.

**Figure 7:**
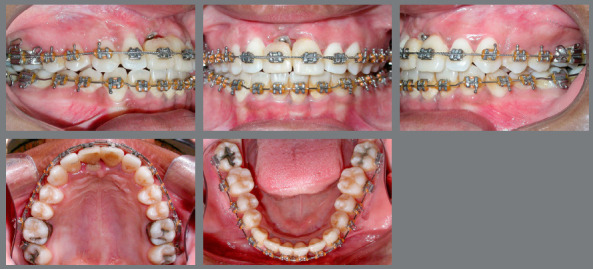
After bone graft in maxillary right central incisor region. Alignment of rotated maxillary second molars with Twist-flex double-archwire mechanics.

**Figure 8:**
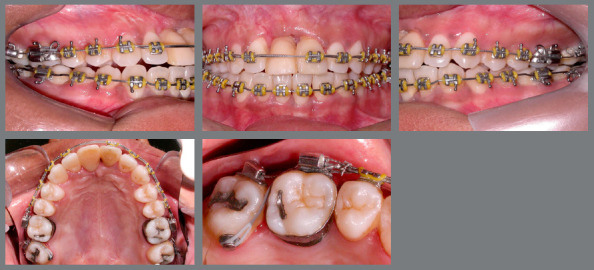
After placement of implant and temporary crown on maxillary right central incisor. Double-archwire mechanics aligning maxillary second molars. Auxiliary mechanics with button and chain elastic, to improve position of the maxillary right second molar.

In the maxillary arch, a 0.019 x 0.026-in stainless steel archwire was inserted, and interproximal reduction was done on maxillary left (#21) and right (#11 provisional crown) central incisors, determining adequate space for rehabilitation. At different surgical times, bone graft surgery was performed in the atrophic region (Fig 7), followed by installation of an osseointegrated implant and implant-supported provisional crown of the #11 (Fig 8).

A crossbite was still observed at the maxillary left and right second molars (#17 and #27), which were also mesially rotated, and did not respond to conventional alignment and leveling. At this point, using a 0.019 x 0.026-in stainless steel archwire as anchorage, double-archwire mechanics was performed with 0.015-in twist-flex followed by 0.014-in stainless steel archwires, together with auxiliary mechanics with button and chain elastic, to improve position of the maxillary right second molar (Figs 7 and 8).

After 34 months of treatment, the fixed appliance was removed, with subsequent placement of a 0.018-in stainless steel wire 3x3 intercanine retainer, bonded to all teeth. A wraparound retainer was used in the maxillary arch. The patient was instructed to wear it full-time for one year, then at night for a year, and finally on alternate nights for another year.

### TREATMENT RESULTS

As result of the orthodontic treatment, there was a great improvement in the smile, which became broad, harmonious and with upright incisors. There was no significant facial changes, with maintenance of the slight deviation of the chin to the right and of the convex profile. A wider palate was noted after the expansion procedure.

**Figure 9:**
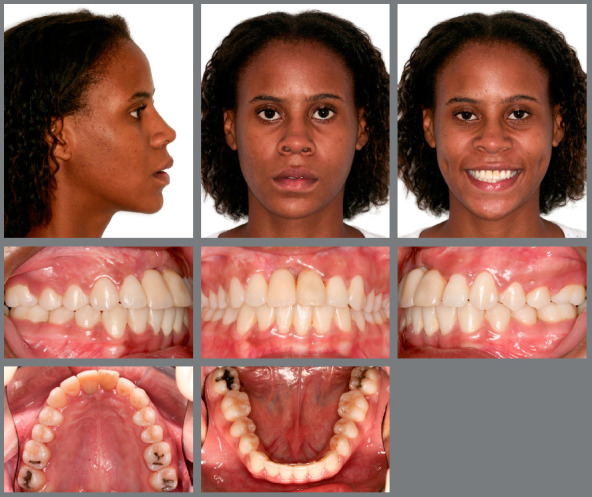
Post-treatment extraoral and intraoral photographs.

Aligned, leveled and coordinated arches were obtained, with good intercuspation in Class I relationship and adequate transverse relationship. A better incisor inclination was achieved, with adequate overjet and overbite correction, and a proper rehabilitation of the maxillary right central incisor (#11) after orthodontic space preparation, surgical procedure and placement of a new temporary crown (Fig 9).

In functional aspects, there was improvement in lip sealing at rest after correction of the maxillary incisors, in addition to obtaining correct excursive guides in laterality and protrusion.

**Figure 10:**
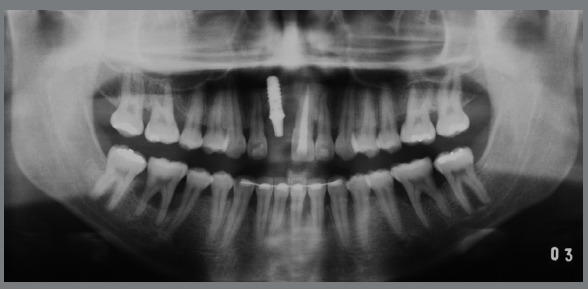
Post-treatment panoramic radiograph.

**Figure 11:**
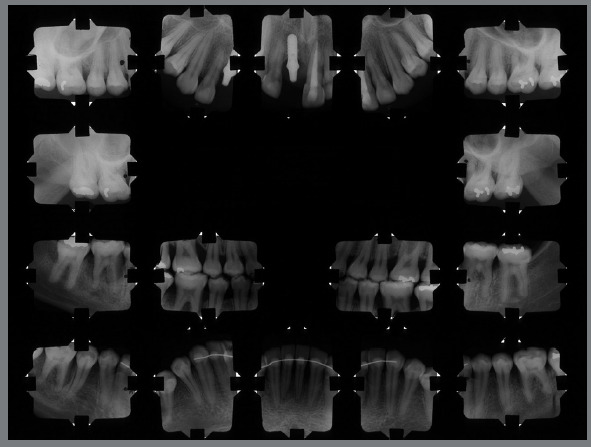
Post-treatment periapical radiographs.

The panoramic radiograph (Fig 10) and complete periapical examination (Fig 11) highlight the generalized bone loss and the regions of the incisors and first molars, most affected by the aggressive periodontitis.

**Figure 12:**
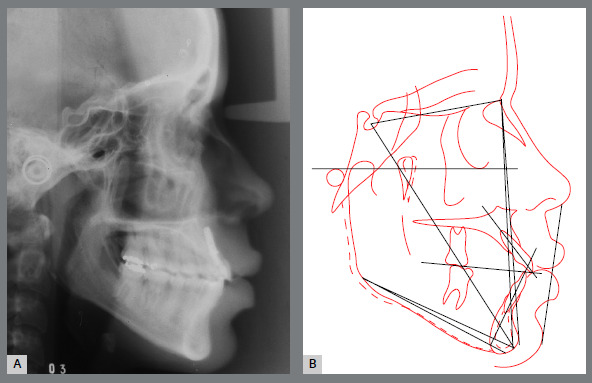
Post-treatment lateral cephalometric radiograph **(**A) and cephalometric tracing **(**B).

**Figure 13:**
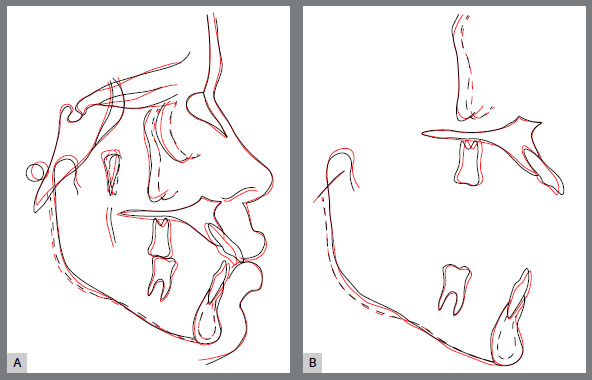
Total **(**A) and partial **(**B) superimpositions of the initial ( black ) and final ( red ) cephalometric tracings

Cephalometric radiograph and cephalometric tracings (Fig 12 and Table 1), as well as cephalometric superimpositions (Fig 13) show that the anteroposterior and vertical skeletal aspect was maintained, with a small reduction in the mandibular plane, without clockwise rotation. There was verticalization of the maxillary incisors and slight projection of the mandibular incisors, with slight molar mesialization in both arches.

**Table 1:** Initial (A) and final (B) cephalometric values.

	MEASURES		Normal	A	B	A/B
Skeletal Pattern	SNA	(Steiner)	82°	84°	84°	0
	SNB	(Steiner)	80°	82°	83°	1
	ANB	(Steiner)	2°	2°	1°	1
	Wits	(Jacobson)	♀ 0 ±2mm ♂ 1 ±2mm	-6.5mm	-5mm	1.5
	Angle of convexity	(Downs)	0°	2°	0°	2
	Y-axis	(Downs)	59°	57.5°	57°	0.5
	Facial Angle	(Downs)	87°	94°	95°	1
	SN.GoGn	(Steiner)	32°	38°	35°	3
	FMA	(Tweed)	25°	30°	28°	2
Dental Pattern	IMPA	(Tweed)	90°	79°	87°	8
	1.NA (degrees)	(Steiner)	22°	37°	32°	5
	1-NA (mm)	(Steiner)	4 mm	13mm	10mm	3
	1.NB (degrees)	(Steiner)	25°	22°	28°	6
	1-NB (mm)	(Steiner)	4mm	6mm	8mm	2
	- Interincisal angle	(Downs)	130°	120°	118°	2
	1-APog	(Ricketts)	1mm	14mm	10mm	4
Profile	Upper Lip - S Line	(Steiner)	0mm	4.5mm	5mm	0.5
	Lower Lip - S Line	(Steiner)	0mm	5.5mm	5mm	0.5

## DISCUSSION

Aggressive periodontitis (AgP) was formerly called juvenile periodontitis or early-onset periodontitis.^2^ Since its introduction in 1999, the term aggressive periodontitis has been the topic of many investigations, but with significant heterogeneity in the understanding and use of this term.^11^ A new periodontitis classification scheme has been adopted in 2018, in which forms of the disease previously recognized as “chronic” or “aggressive” are grouped under a single category (“periodontitis”) and are further characterized based on a multi-dimensional staging and grading system.^12^ On the other side, recent studies^3^ ^,^ ^4^ ^,^ ^6^ ^,^ ^11^ ^,^ ^13^ have continued to adopt the term aggressive periodontitis describing research samples and case reports involving orthodontic-periodontal treatments.

The general approach to determining the orthodontic diagnosis and treatment plan in adults with periodontitis requires an examination of the periodontal status, such as: prevalence of periodontal pockets, degree of tooth mobility, extent of tooth loss, and behavioral factors. In addition to conventional periodontal disease screening, blood IgG antibody titer test and microbiologic monitoring of periodontal pathogens may quantitatively evaluate periodontitis.^14^

Haas et al.^15^ highlighted that clinicians are frequently uncertain about how AgP patients will respond to periodontal treatment. They studied the predictors of clinical outcomes after periodontal treatment of AgP in a 12-month randomized trial. Patient- and tooth-related factors predicting better treatment outcomes of AgP included extent of periodontitis, use of azithromycin, type of teeth affected, plaque, and baseline periodontal probing depth.

A systematic review with meta-analysis showed that in 26 patients without periodontitis and in 69 periodontitis treated patients, minimal changes in periodontal outcomes were reported after orthodontic therapy. They concluded that, based on a small number of low-quality studies (n = 26), orthodontic treatment had no significant impact on periodontal outcomes in non-periodontitis and in stable periodontitis treated patients.^16^

Carvalho et al.^13^ showed that orthodontic treatment can be performed in AgP patients, and observed that orthodontic movement of the teeth resulted not only in stability of the periodontal tissues, but also in a slight but significant improvement in periodontal conditions. They also concluded that AgP patients with reduced periodontium can undergo orthodontic movement without additional attachment loss, but rigid biofilm control by the patients and regular professional follow-up visits are necessary. 

According to Castellanos-Cosano et al.^7^, a comprehensive periodontal treatment needs to take place before other intervention, and periodontal maintenance and follow-up throughout the orthodontic treatment and after plays a crucial role. For Levin et al.^17^, in patients who do not comply with the required oral hygiene, active orthodontic treatment should be postponed until satisfactory plaque control is achieved. Periodontal recall appointments once every three months are advised for the period of active orthodontic treatment, and this should be performed in a separate dedicated visit.

The treatment of pathologic extruded and flared anterior teeth is a main concern in AgP patients,^18^ and early diagnosis and treatment are essential for successful long-term prognosis.^17^ Combining periodontal therapy, orthodontic treatment, and prosthodontics can greatly improve function and the esthetic result.^7^ It is also important to highlight that there are unique aspects in the orthodontic retention in these cases.^10^

As general limitations of the treatment plan in the present case, it was very dependent on the patient’s cooperation in terms of excellent oral hygiene and assiduity in periodontal follow-up, as well as the success of the multidisciplinary treatment in the rehabilitation of the maxillary anterior region. The periodontal condition brought unpredictability to the case, as some teeth could present excessive mobility, requiring interruption of the application of orthodontic force and changes in the treatment plan.

The prognosis could be considered uncertain, especially for some regions that already had more severe bone loss, with the risk of worsening the problem; as well as for long-term stability, due to the unpredictability of the patient’s periodontal health, which would require constant periodontal monitoring.

Despite the great periodontal limitations of the present case, it was believed that leaving this patient without any orthodontic treatment could be more harmful in the long term than taking the risk of aggravating the periodontal condition of some regions, for example teeth #36 and #46. Post-treatment radiographs suggest that there was no worsening of the general and localized periodontal aspect, but limitations can be observed in obtaining correct root parallelism, due to bone deficiency. Shen et al.^19^ showed that, after active periodontal treatment, orthodontic treatment in AgP patients had not aggravated inflammation and alveolar bone resorption, but root resorption occurred in two-thirds of incisors, approximately. Otherwise, we did not observe significant root resorption in the present clinical case.

It is expected that the patient’s new occlusal, clinical and aesthetic condition will contribute to the maintenance of better oral health. Unfortunately, it was not possible to recall the present patient for post-retention follow-up records. Zafiropoulos et al.^20^ described the 7-year result of periodontal maintenance after a complex orthodontic-periodontal treatment in a case of generalized AgP. They concluded that a consistent resolution of generalized AgP could be achieved utilizing a combined mechanical and antimicrobial treatment, followed by periodontal maintenance and microbiological monitoring.

The total time of active orthodontic treatment was influenced by the multidisciplinary aspect of the present case, as there were several visits for routine periodontal control, more invasive periodontal procedures, surgeries at different times for grafting and subsequent implant placement in the #11 region, in addition to the prosthetic finishing.

## CONCLUSIONS

Orthodontic treatment with adequate occlusal, functional and esthetic results can be performed in young adults presenting aggressive periodontitis. An interdisciplinary approach is required, with frequent periodontal follow-up before, during and after orthodontic treatment, allowing the correction of dental positions without aggravating bone loss. 
